# Participation Dynamics in Population-Based Longitudinal HIV Surveillance in Rural South Africa

**DOI:** 10.1371/journal.pone.0123345

**Published:** 2015-04-13

**Authors:** Joseph Larmarange, Joël Mossong, Till Bärnighausen, Marie Louise Newell

**Affiliations:** 1 Centre Population & Développement (UMR 196 Paris Descartes Ined IRD), Institut de Recherche pour le Développement, Paris, France; 2 Africa Centre for Health and Population Studies, University of KwaZulu-Natal, Mtubatuba, South Africa; 3 Surveillance & Epidemiology of Infectious Diseases, Laboratoire National de Santé, Luxembourg, Luxembourg; 4 Department of Global Health and Population, Harvard School of Public Health, Harvard University, Boston, Massachusetts, United States of America; 5 Faculty of Medicine, Faculty of Social and Human Sciences, University of Southampton, Southampton, United Kingdom; FIOCRUZ, BRAZIL

## Abstract

Population-based HIV surveillance is crucial to inform understanding of the HIV pandemic and evaluate HIV interventions, but little is known about longitudinal participation patterns in such settings. We investigated the dynamics of longitudinal participation patterns in a high HIV prevalence surveillance setting in rural South Africa between 2003 and 2012, taking into account demographic dynamics. At any given survey round, 22,708 to 30,495 persons were eligible. Although the yearly participation rates were relatively modest (26% to 46%), cumulative rates increased substantially with multiple recruitment opportunities: 68% of eligible persons participated at least once, 48% at least twice and 31% at least three times after five survey rounds. We identified two types of study fatigue: at the individual level, contact and consent rates decreased with multiple recruitment opportunities and, at the population level, these rates also decreased over calendar time, independently of multiple recruitment opportunities. Using sequence analysis and hierarchical clustering, we identified three broad individual participation profiles: consenters (20%), switchers (43%) and refusers (37%). Men were over represented among refusers, women among consenters, and temporary non-residents among switchers. The specific subgroup of persons who were systemically not contacted or refusers constitutes a challenge for population-based surveillance and interventions.

## Introduction

Over the past two decades, population-based longitudinal HIV surveillance [1–4] has proven to be of great value in helping to understand the dynamics of the HIV pandemic and to evaluate the impact of HIV programs in developing countries [5–7]. HIV incidence has commonly been inferred from observed prevalence changes over time in subsequent cross-sectional surveys (such as Demographic and Health Surveys) using some mortality assumptions [8,9]. However, with access to antiretroviral treatment expanding rapidly, such approaches are becoming increasingly unreliable due the rapidly decreasing HIV-specific mortality [6,10–12]. Population-based longitudinal HIV surveillance therefore remains the gold standard for measuring HIV incidence and monitoring incidence changes over time [13].

However, little is known about the underlying dynamics of participation patterns (i.e. participation outcomes in a sequence of survey rounds) in HIV surveillance, which are embedded within demographic dynamics (mortality, aging in, migration) and how these participation dynamics affect HIV prevalence and incidence estimates. Previous work has mainly focused on factors associated with participation in single, or at best two cross-sectional surveys [14–18], or on cumulative rates in HIV negative people to estimate incidence [19]. Participation in a longitudinal context is a dynamic process (i.e. acceptability could change over time), which interacts with underlying demographic changes.

Furthermore, universal repeat HIV testing and immediate antiretroviral treatment (“test and treat”) strategies constitute one of the major current research questions in the HIV field [20], with four on-going large-scale cluster randomized trials [21–25] in Southern Africa. These trials face similar operational challenges in terms of contact and participation at population level [26].Understanding participation patterns in the context of a longitudinal HIV surveillance could be informative for these trials and any population-based intervention.

One of the main challenges facing population-based HIV surveillance is to ensure adequate statistical representation of the general population, or, at the very least, adequate statistical representation of the general population within strata of key variables used to re-weight the surveyed population to reflect the general population. A high participation rate is required for accurate estimation of HIV prevalence, as participation is likely to depend on HIV status [27,28] which leads to biased estimates [29–31]. Further, because of the longitudinal nature of HIV surveillance, repeat participation and attrition rates are crucial to both ensure comparability of prevalence estimates over time and to measure HIV incidence, because incidence estimation requires at least two measurement points in time [32].

The main purpose of our study is thus to measure the evolution of annual participation rates, to identify potential study ‘fatigue’ over time, to investigate longitudinal participation patterns and to assess their potential impact on estimates of HIV prevalence and incidence in a high HIV prevalence surveillance setting in a rural area in KwaZulu-Natal, South Africa, where annual HIV surveys have been conducted since 2003 among the adult resident population.

## Material and Methods

### Study setting

The Africa Centre for Health and Population Studies has hosted a socio-demographic household surveillance in a rural sub-district of uMkhanyakude in northern KwaZulu-Natal (South Africa) since 2000. The surveillance area is 438 km^2^ in size and includes a population of approximately 90 000 isiZulu-speaking people [1]. The study area is characterized by high adult HIV prevalence (24% in adults aged 15 years and older in 2011) and high levels of poverty and unemployment (in 2010, 67% of adults over the age of 18 were unemployed). At any point in time, about one-third of the population under demographic surveillance does not physically reside in the surveillance area, although they are considered to be household members (membership is defined by household head). Demographic events and socio-economic data are collected on a regular basis from a key household informant.

Starting in 2003, a nested HIV surveillance was conducted among resident adults, who were invited to respond to a health and sexual behaviour questionnaire and to provide a small blood sample which was tested for HIV [14]. For each survey round, teams of two trained fieldworkers visited each eligible individual in their households. If a person was absent, the field workers made up to four repeat visits to the same household. Except for the first HIV surveillance round, which took place over 18 months starting in 2003, all subsequent rounds were done on annual basis. For this analysis, we used data collected between 2003 and 2012, corresponding to a total of nine survey rounds.

### Eligibility to participate in HIV surveillance

All adults residing in the area and who were able to provide informed written consent were eligible to participate in HIV surveillance. From 2003 to 2006, eligibility was restricted to women aged 15–49 years and men aged 15–54. From 2007 onwards, all persons aged 15 years and older were eligible.

The population of eligible resident participants changes substantially on a yearly basis. Thus from 2004 onwards, at the beginning of each calendar year, a list of all resident persons eligible to participate in HIV surveillance was generated from information available in the demographic database. During fieldwork operations, some persons became ineligible “a posteriori”, because the information of death, out-migration or sickness was not available at the time the eligibility list was generated or because their situation changed. These persons were treated as ineligible for the purpose of our analysis.

### Ethic Statement

Informed written consent was obtained from all adult eligible persons aged 15 year or older for participation in the surveillance and to provide a small blood sample for HIV analysis for research purposes. As permitted by the regulatory framework governing research in South Africa at the time of the study [33], we obtained written informed consent from adolescents aged 15–17 years themselves. Ethical approval for HIV surveillance (reference numbers E029/03, E122/06 for the extension beyond age 50 years and BF233/09 for the combined ages) was obtained from the Biomedical Research Ethics Committee (BREC) of the University of KwaZulu-Natal, and renewed on an annual basis. The BREC was aware that some of the surveillance participants were minors and approved the age range of participation and the specific consent procedure for minors.

### Data Availability Statement

Data are available from the INDEPTH data repository (doi: 10.7796/INDEPTH.AC.HIV.Participation.2003.2012.v1, http://www.indepth-ishare.org/index.php/catalog/53/).

### Annual participation rate

In any given survey round, we defined the HIV surveillance acceptance rate as the proportion consenting to provide a blood sample for HIV testing among persons who were contacted; and the effective HIV surveillance coverage as the proportion consenting to provide a blood sample among all eligible persons.

### Statistical analysis

The use of sequence analysis [34,35] in social sciences has significantly increased during the last two decades [36,37]. In this approach, outcomes are not studied independently of each other but rather as a sequence. For our study, we considered participation as a sequence of successive participation outcomes, of which there were three types: (i) the person was not contacted; (ii) the person was contacted but refused to participate; (iii) the person was contacted and consented to participate, i.e. the person accepted to provide a finger prick blood sample for HIV testing.

**Fig 1:**
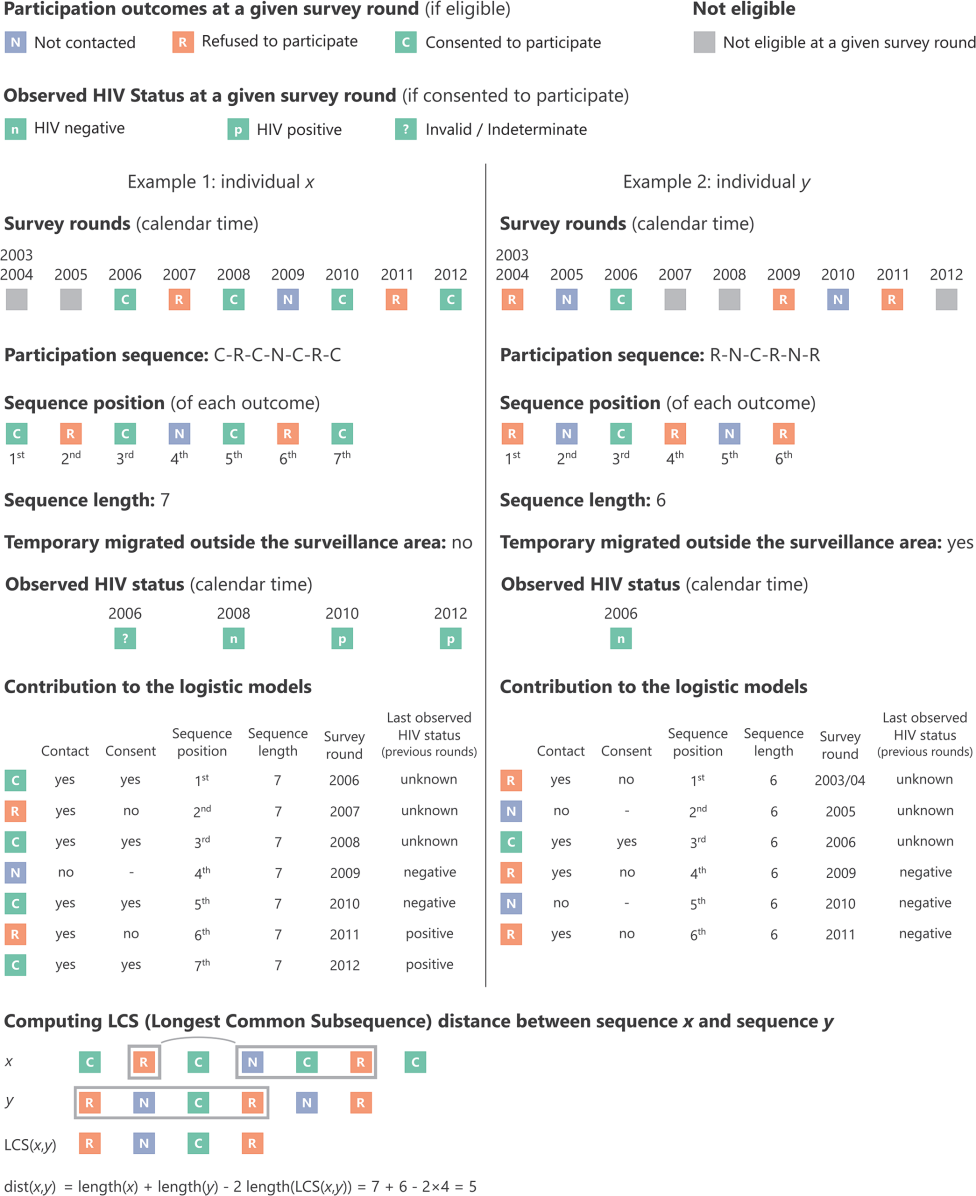
Examples of the methodological approach used to compute sequences and Longest Common Subsequence distance.

The participation sequence of a person, as illustrated on Fig 1, is the series of participation outcomes for each surveillance round during which she/he was eligible for HIV surveillance. The length of a participation sequence is the total number of times (minimum: 1; maximum: 9) a person was eligible for HIV surveillance, i.e. the number of participation outcomes for that person. The length of the participation sequence can vary between persons. For example, due to temporary outmigration, a participant could become ineligible for one (or more) surveillance round in between two rounds during which she/he was eligible (see example 2 on Fig 1). The participation sequence length is also influenced by the year in which a person first becomes eligible, or ceases to be eligible due to outmigration or death. Thus, a person who was 15 years old in 2010 could only have a maximum participation sequence length of three by the end 2012. The sequence position refers to the position of a given participation outcome within a specific sequence.

To investigate the concept of ‘study fatigue’, which postulates that persons become less interested and thus less likely to participate repeatedly in a study over time, we used two binomial logistic regression models to estimate the probability of being contacted vs. not being contacted (using all participation outcomes, see Fig 1) and the probability of consenting vs. refusing to participate (among contacts), as a function of sequence position (i.e. the n^th^ time where a person was eligible), while adjusting for sex, age, sequence length, survey round (calendar time), having temporarily migrating outside the study area and prior observed HIV status.

To investigate patterns of participation over the long term, we focused our analysis on persons with a long participation sequence length, i.e. persons who were eligible at least seven times. We restricted the sequence analysis to long sequences (of length of 7 to 9) because the distance metric between two sequences we employed is also influenced by difference in sequence length. A hierarchical classification of the entire population would have been unduly influenced by sequence length rather than participation status dynamics. Dissimilarity between sequences was calculated using optimal matching techniques before dividing the population into several groups (participation profiles) using hierarchical classification and describing participation sequences classes. We used the Longest Common Subsequence (LCS) distance to compute distances between sequences (see example on Fig 1). LCS distance is a special case of optimal matching distance [38]. A dendrogram was computed using the Wald algorithm. We used the higher relative loss of inertia criterion to determine the optimal number of partitions (see function HCPC in FactoMineR package [39,40]). We used odds ratio as effect size indicator to describe participation profiles.

Sankey diagrams (flow diagrams in which the thickness of the lines is proportional to the flow quantity) were used to represent the dynamic of the eligible population over time and the participation dynamic by sequence position of each participation profile.

All statistical analyses were performed using R 3.0.1 [41]. Sankey diagrams were constructed using an adapted version of Mike Bostock’s script for D3.js (http://bost.ocks.org/mike/sankey/). Sequence analysis was performed using the TraMineR package [42]. Color palettes were chosen from http://colorbrewer2.org/ to be colorblind safe. All figures were edited with Inkscape 0.48.

## Results

### Population dynamics

**Fig 2:**
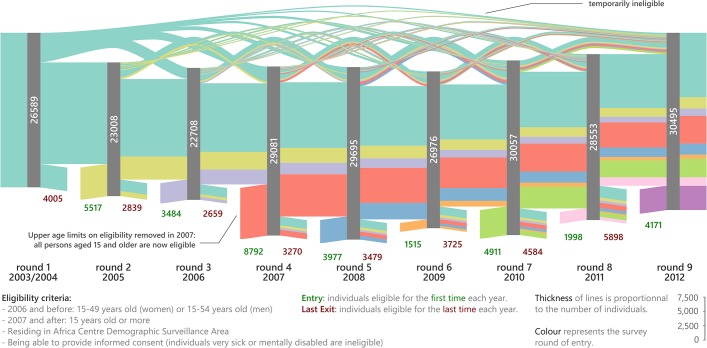
Eligibility flow chart in a Sankey diagram (flow diagram in which the thickness of the lines is proportional to the flow quantity) by survey round. Different colours represent the year in which persons first became eligible for HIV surveillance. The numbers in the grey bars represent the number of persons eligible for HIV surveillance during a given survey round. The numbers below the grey bars represent the persons who enter or exit the HIV surveillance eligible population because of death, in- and out-migration or ageing into the open cohort.Annual participation rates and cumulative rates.

Fig 2 shows the longitudinal eligibility dynamics of the population of individuals under HIV surveillance. Whereas cumulatively 60,954 persons were eligible at least once over the decade (S1 Table), only between 22,708 and 30,495 persons were eligible during any single survey round. Between 2,839 and 5,898 adults definitively exited (deaths, out-migration, before 2007 becoming age-ineligible) and between 1,515 and 8,792 adults entered into (ageing into, in-migration) the HIV surveillance in single survey rounds. The number of entries was higher from 2007 onwards because the upper-age eligibility criterion (which was 50 for women and 55 for men prior to 2007) was removed. More than a quarter of all eligible persons (28.8%) were temporarily ineligible during at least one round, due to temporary out-migration. Of the 26,589 persons that were eligible in the first round in 2003/2004, only 11,088 (41.7%) remained eligible in round 9 in 2012. Taken together, these data suggest a fairly rapid turnover of the underlying population under HIV surveillance in this setting due to demographic processes including migration, ageing, and death.

**Fig 3:**
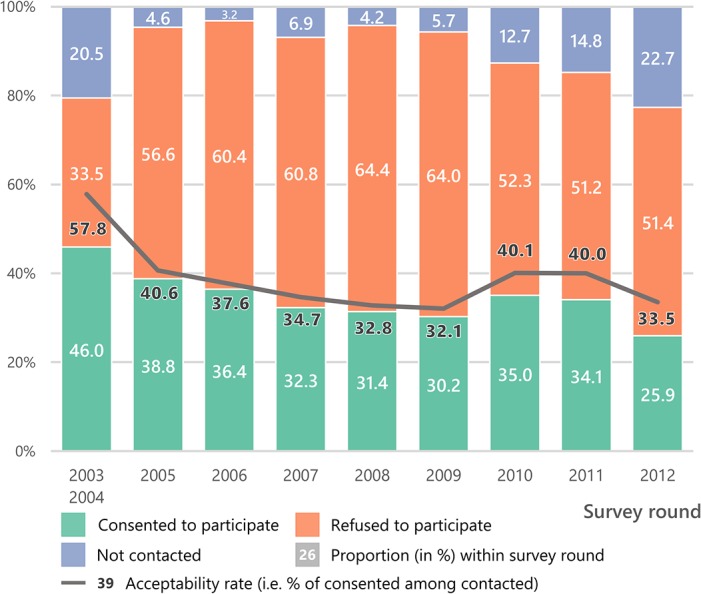
Crude annual participation rates (including the effective HIV surveillance coverage) and HIV surveillance acceptance rates by survey round.

Overall, the HIV surveillance acceptance rate remained relatively stable at 32–41% after the first survey round (Fig 3). The effective HIV surveillance coverage decreased from 46% in 2003/2004 to 26% in 2012, reflecting changes in contact rates. Between 2003/2004 and 2005, the proportion not contacted decreased from 21% to 5%, due to logistic improvements in the way the survey was conducted by establishing a list of eligible persons at the beginning of the survey round (see materials and methods). However, between 2005 and 2012, the proportion not re-contacted increased progressively from 5% to 23%, indicating potential ‘study fatigue’.

**Fig 4:**
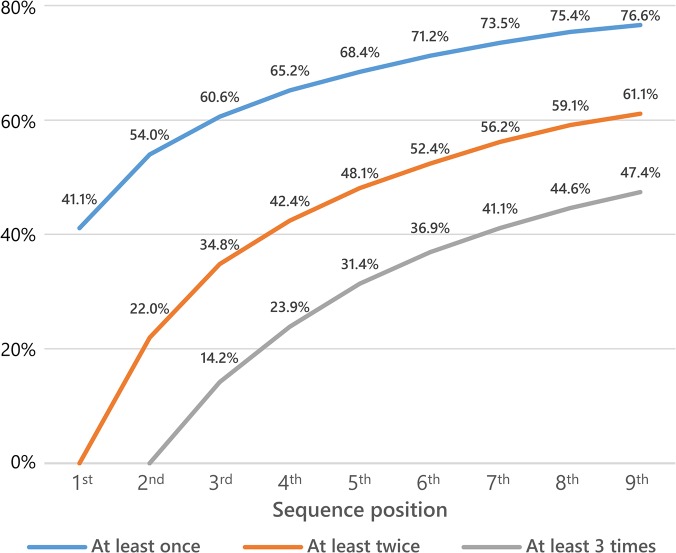
Proportion of persons having consented to provide a blood sample at least once, twice or three times as a function of sequence position.

Although the effective HIV surveillance participation rate in any single survey round might appear to be low, it is important to consider multiple recruitment opportunities in a longitudinal setting. Thus, cumulatively after five rounds, 68% of all eligible persons participated at least once, 48% at least twice and 31% at least three times (Fig 4). Due to the fact that a minimum of one blood sample is required for estimating HIV prevalence and two blood samples for HIV incidence, only two-thirds of the sample was used for prevalence and half of the sample for incidence estimation.

### Study ‘fatigue’

The concept of study ‘fatigue’ postulates that persons become less likely to participate repeatedly in a study over time. Overall, we found that men and persons aged 20–49 years were less likely to be contacted or to consent to participate if contacted than women (p<0.001) and persons of other ages (Table 1). Persons who had never consented to provide a blood sample were less likely to consent in subsequent rounds (p<0.0001). Having a prior positive HIV test was significantly associated with refusal (p<0.0001), but was not associated with non-contact (p = 0.8965). Sequence length was negatively associated with contact (adjusted Odds Ratio [aOR] length of 9 vs. length of 1: 2.955, p<0.0001) and, to a lesser extent, with refusal (aOR length of 9 vs. length of 1: 1.142, p<0.0001). Persons having temporarily migrated outside the surveillance area were less likely to be contacted (aOR = 0.636, p<0.0001) but not to refuse to participate (aOR = 0.986, p = 0.2094). The sequence position was negatively associated with contact and with consent, which could indicate a certain degree of study fatigue (aOR 9^th^ outcome vs.1^st^ outcome is 0.302, p<0.0001 for contact and 0.178, p<0.0001 for consent). While contact improved in the first survey rounds, calendar time was found to be negatively associated with contact in later rounds (aOR 2012 vs. 2008: 0.206, p<0.0001). For consent, adjusted odds ratios decreased between 2003/2004 and 2008, followed by an improvement in 2010 and 2011 (aOR vs. 2008: 1.508 and 1.472 respectively, p<0.0001).

**Table 1:** Factors associated with contact and consent from two binomial logistic regression model.

	Contacted vs. Not contacted			Consented vs. Refused (among contacted)		
	aOR	p		aOR	p	
**Sequence position**						
1^st^	1			1		
2^nd^	0.910	0.0028	**	0.411	0.0000	***
3^rd^	0.704	0.0000	***	0.290	0.0000	***
4^th^	0.654	0.0000	***	0.249	0.0000	***
5^th^	0.591	0.0000	***	0.240	0.0000	***
6^th^	0.518	0.0000	***	0.217	0.0000	***
7^th^	0.450	0.0000	***	0.199	0.0000	***
8^th^	0.392	0.0000	***	0.196	0.0000	***
9^th^	0.302	0.0000	***	0.178	0.0000	***
**Sex of participant**						
female	1			1		
male	0.576	0.0000	***	0.722	0.0000	***
**Age of participant**						
15–19	1.589	0.0000	***	1.326	0.0000	***
20–29	1	0		1		
30–39	0.801	0.0000	***	0.908	0.0000	***
40–49	0.853	0.0000	***	1.023	0.1531	-
50 or more	1.490	0.0000	***	1.425	0.0000	***
**Sequence length**						
1	1			1		
2	0.981	0.5763	-	1.043	0.1335	-
3	1.448	0.0000	***	1.087	0.0018	**
4	1.655	0.0000	***	1.087	0.0030	**
5	1.959	0.0000	***	1.107	0.0000	***
6	2.503	0.0000	***	1.164	0.0000	***
7	2.473	0.0000	***	1.121	0.0001	***
8	2.784	0.0000	***	1.154	0.0000	***
9	2.955	0.0000	***	1.142	0.0000	***
**Survey round**						
2003/2004	0.186	0.0000	***	2.650	0.0000	***
2005	0.879	0.0049	**	1.332	0.0000	***
2006	1.390	0.0000	***	1.283	0.0000	***
2007	0.564	0.0000	***	1.029	0.1596	-
2008	1			1		
2009	0.759	0.0000	***	1.024	0.2438	-
2010	0.339	0.0000	***	1.508	0.0000	***
2011	0.305	0.0000	***	1.472	0.0000	***
2012	0.206	0.0000	***	1.077	0.0013	**
**Last observed HIV status (in previous rounds)**						
negative	1			1		
positive	1.003	0.8965	-	0.919	0.0000	***
unknown	0.770	0.0000	***	0.196	0.0000	***
**Having temporarily migrated outside the surveillance area**						
no	1			1		
yes	0.636	0.0000	***	0.986	0.2094	-

247,040 observations for model 1 (contacted vs. not contacted). 220,096 observations for model 2 (consented vs. refused). aOR: adjusted Odds Ratio.

*** p ≤ 0.001,

** 0.001< p ≤ 0.01,

* 0.01 < p ≤ 0.05,—p > 0.05.

### Participation profiles

**Fig 5:**
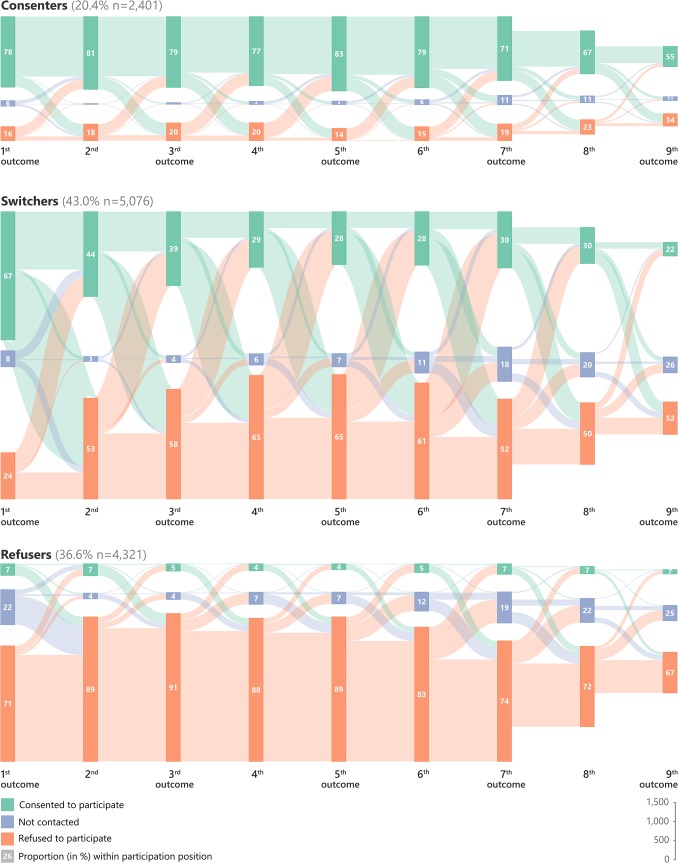
Participation flow chart by sequence position in a Sankey diagram (flow diagram in which the thickness of the lines is proportional to the flow quantity) of the three participation profiles (consenters, switchers, refusers) identified by the cluster analysis in persons with long participation sequence (7–9 rounds). This flowchart represents the flows between participation outcomes from one sequence position to the next one.

In order to identify participation profiles, we conducted a cluster analysis of persons with long participation sequences, with a length of 7–9 survey rounds. While persons with long (7–9 rounds) participation sequences represented 19% of the 60,954 persons who were ever eligible, they represented 33–47% of eligible persons during any given survey round (S1 Fig) and 38.3% of all participation outcomes. Hierarchal clustering analysis suggests three major classes of participation profiles (S2 and S3 Figs) corresponding to three broad participation types. The participation flowchart of each profile is represented in Fig 5 and characteristics described in Table 2. For convenience, and according to their participation flowchart (Fig 5), we decided to name these participation profiles as “consenters” (20% of long sequences), “switchers” (43%) and “refusers” (37%).

**Table 2:** Characteristics of three participation profiles (consenters, switchers, refusers) identified by the cluster analysis in persons with long participation sequence (7–9 rounds).

	Consenters	Switchers	Refusers	All
n	2,401	5,076	4,321	11,798
%	20.4	43.0	36.6	100.0
**Sex**				
female (%)	75.0	62.0	56.7	62.7
male (%)	25.0	38.0	43.3	37.3
**Age at first participation**				
15–19 y. (%)	24.6	27.3	17.2	23.0
20–29 y. (%)	19.3	24.2	23.3	22.9
30–39 y. (%)	19.4	21.0	28.3	23.4
40–49 y. (%)	30.6	22.5	26.1	25.5
50+ y. (%)	6.1	5.0	5.0	5.2
median (years)	33	29	33	31
**HIV tests**				
mean	6.2	2.9	0.5	2.7
At least one (%)	100.0	99.9	35.0	76.2
At least two (%)	100.0	84.1	9.8	60.1
At least three (%)	100.0	58.2	1.3	45.9
**Observed HIV prevalence by sequence position (%)**				
1^st^	15.5	18.5	22.1	17.7
2^nd^	16.7	22.6	24.7	20.2
3^rd^	17.9	24.2	28.6	21.5
4^th^	20.9	26.6	28.6	23.7
5^th^	23.9	29.6	28.7	26.4
6^th^	26.3	31.6	30.3	28.7
7^th^	27.7	36.8	38.8	32.5
8^th^	27.2	35.4	40.3	31.7
9^th^	24.5	31.6	42.9	29.1
**Observed HIV seroconversions**				
number of conversions	275	448	24	747
person-years of observation	13,345	16,064	1,068	30,477
observed HIV incidence (‰)	20.6	27.9	22.5	24.5
**Having temporarily migrated outside the surveillance area**				
no	61.9	55.5	60.5	58.6
yes	38.1	44.5	39.5	41.4

Consenters generally had refusal rates below 20% at any sequence position (except for the 8^th^ and the 9^th^ outcome where it increased to 23% and 34%, respectively). Consenters who refused to participate in any round were more likely to consent to participate in the subsequent round (Fig 5). On average, consenters participated more than 6 times and all consenters participated at least three times (Table 2). On the other hand, refusers participated 0.5 times on average, 35% participated at least once and 10% at least twice. Switchers displayed important patterns of switching between consent and refusal (Fig 5). The acceptance rate among switchers decreased progressively from 67% for first outcome to 22% for the 9^th^ outcome. Almost all of the switchers (99.9%) were tested at least once and 84% at least twice, the average number of HIV tests being 2.9 (Table 2).

Men were over-represented among refusers, women among consenters, and temporarily ineligible persons among switchers (Table 2). Over-represented age-groups for each participation profile were 15–29 years old for switchers, 20–49 for refusers and 15–19 and the 40 years and older for consenters. Observed HIV incidence was higher among switchers than consenters (2.8% versus 2.1%, OR = 1.353). Most importantly, observed HIV prevalence by sequence position was higher among refusers than among consenters (22.1% vs. 15.5%, OR = 1.553 for the first outcome; 42.9% vs. 24.5%, OR = 2.313 for the 9^th^ outcome) or among switchers (22.1% vs. 18.5%, OR = 1.254 for the first outcome; 42.9% vs. 31.6%, OR = 1.624 for the 9^th^ outcome).

Observed HIV incidence among refusers (2.3%) was estimated only on the 9.8% who provided a sample for testing twice and is thus a limited indicator for this group (not representative and sample size too small, there is no statistically significant difference between observed incidence among refusers and incidence among consenters, p = 0.7643, or switchers, p = 0.3418, using Fisher test). Another proxy of HIV incidence could be the increase of HIV prevalence. Between the first and the 9^th^ outcomes, observed HIV prevalence increased by 9.0% for consenters, 13.1% for switchers and by 20.8% for refusers. It should be noted however that prevalence could also increase in case of improved coverage of HIV treatment and, consequently, of reduction in mortality due to HIV.

## Discussion

Due to substantial levels of mortality [6], in- and out-migration [43,44], ageing into the cohort and the fact that migration is associated with HIV and death [45,46], the target population of HIV surveillance in this setting changes considerably on a yearly basis. For example overall 21% of the target population was eligible only once and 29% were temporarily ineligible.

One of the main criticisms addressed at population-based HIV surveillance is the low yearly participation rates [47]. Our analysis shows that the proportion of persons who were effectively covered by HIV surveillance increased substantially with cumulative recruitment opportunities. Approximately two-thirds consented at least once and approximately half at least twice to participate within five years of becoming eligible.

Refusal could constitute an important source of bias for prevalence and incidence estimation. We showed that refusal was associated with sex and age, and we estimated HIV prevalence to be substantially higher among refusers [48,49]. Of concern is the finding that HIV incidence is likely to be higher among refusers. Our results are consistent with other analyses showing that HIV-infected persons were significantly less likely than HIV-uninfected persons to consent to participate during a single survey round [17]. Persons knowing that they are HIV positive could be reluctant to participate in the surveillance as the Africa Centre is known to collaborate with the Department of Health in the local HIV treatment and care programme [50], but also because they may think that their status is already known and thus another test would be futile [18]. Consequently, HIV prevalence estimates from cross-sectional surveys would under-estimate the true HIV prevalence in the population, because persons enrolled in pre-antiretroviral treatment (ART) care or on ART care would less likely participate in HIV surveillance. This challenge could be addressed by asking persons who do not consent to give blood to report their HIV status or whether they are on pre-ART care or in care, although this could pose ethical challenges [51].

We were able to detect two types of study ‘fatigue’. The first was clearly related to individual-level ‘fatigue’, i.e. the more frequently persons were asked to participate, the less likely they were to repeatedly consent. Secondly, we also detected a wider ‘surveillance system fatigue’, i.e. a tendency over time for participation rates to decrease in later rounds compared to earlier rounds. This could be due to a changing local social perception of HIV surveillance, of persons not being convinced of the utility of HIV surveillance or even of a fatigue of the surveillance system including field workers. The latter could explain the observed increase of non-contacts in later survey rounds. However, qualitative work would be required to explore these hypotheses.

From an HIV intervention perspective, our results could be used to help assess the likely population impact of a test and treat strategy currently evaluated through large trials. One of the main challenges of test and treat trials is to repeatedly reach a high proportion of the total population. Even if the setting of a trial is different from a surveillance setting, our study shows that refusal and non-contacts could be a major issue. More than a fifth (23.4%, see Fig 3) of the total population was never tested after nine survey rounds. A similar result has been shown in the context of home-based testing in Malawi where 11% of individuals remained unreached after two home-based testing campaigns [52]. Preliminary results (after three home-based survey rounds) from the ANRS 12249 Treatment as Prevention trial, currently on-going in the same district as our HIV surveillance, showed a contact rate per survey round of 78% and a HIV ascertainment rate per survey round of 82% [26]. Although these rates are better than the ones observed in our study, our results highlight the risk that a study fatigue may occur over time.

To our knowledge, this is the first time that Sankey diagrams and this type of sequence analysis has been applied to longitudinal HIV epidemiology. These methods allow a detailed description of the complex participation dynamics in an open longitudinal cohort and deserve wider attention in similar other settings.

## Supporting Information

S1 Table Distribution of participants by sequence length. (PDF)

S1 Fig Eligibility flow chart in a Sankey diagram (flow diagram in which the thickness of the lines is proportional to the flow quantity) by survey round and sequence length (represented by colours). The numbers in the grey bars represent the number of persons eligible for HIV surveillance during that round. The numbers below the grey bar represents the persons who enter (green) or exit (red) the HIV surveillance because of death, migration or ageing into the open cohort. Sequence length corresponds to the total number of times a person has been eligible for HIV surveillance. Individual sequences have been categorized as short (length of 1 to 3), mid (length of 4 to 6) or long (length of 7 to 9).
(TIFF)

S2 Fig Growth of inertia by number of classes (hierarchical classification, participation sequences of length 7–9). (TIFF)

S3 Fig Dendrogram of the LCS distance between participation sequences, index plot of participation sequences of length 7–9 and partition in three participation profiles (consenters, switchers, refusers). A dendrogram is a tree diagram illustrating the arrangement of the participation sequences produced by hierarchical clustering. Partition is obtained by cutting the dendrogram at a specific height and is represented by red rectangles. Profiles have been named according to their participation pattern (see Fig 5).
(TIFF)
