# Supplementary material for: Participation Dynamics in Population-Based Longitudinal HIV Surveillance in Rural South Africa
Source: PLoS One. 2015 Apr 13;10(4):e0123345. doi: 10.1371/journal.pone.0123345 (PMC4395370; doi:10.1371/journal.pone.0123345)
Supplement: S1 Table — (PDF) [file pone.0123345.s001.pdf]

S1 Table. Distribution of participants by sequence length

Sequence length	Frequency	Proportion of person (%)	Proportion of eligibilities (%)
1	13,042	21.4	5.3
2	8,455	13.9	6.8
3	8,568	14.1	10.4
4	5,837	9.6	9.4
5	6,151	10.1	12.4
6	7,103	11.7	17.2
7	3,843	6.3	10.9
8	3,711	6.1	12.0
9	4,244	7.0	15.5
Total	60,954	100.0	100.0
