# Supplementary figures and images for: Participation Dynamics in Population-Based Longitudinal HIV Surveillance in Rural South Africa
Source: PLoS One. 2015 Apr 13;10(4):e0123345. doi: 10.1371/journal.pone.0123345 (PMC4395370; doi:10.1371/journal.pone.0123345)

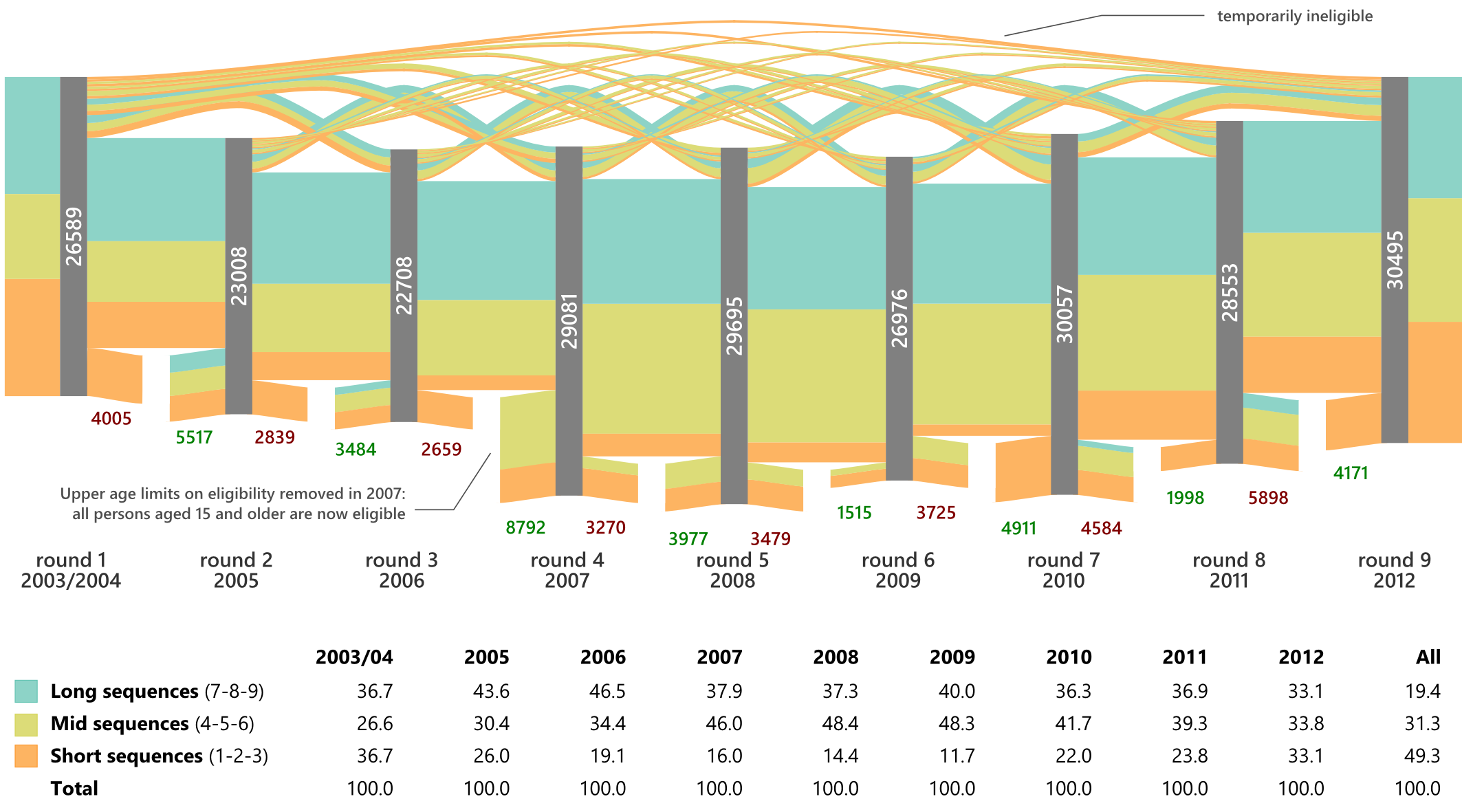

Supplement: S1 Fig — The numbers in the grey bars represent the number of persons eligible for HIV surveillance during that round. The numbers below the grey bar represents the persons who enter (green) or exit (red) the HIV surveillance because of death, migration or ageing into the open cohort. Sequence length corresponds to the total number of times a person has been eligible for HIV surveillance. Individual sequences have been categorized as short (length of 1 to 3), mid (length of 4 to 6) or long (length of 7 to 9). (TIFF) [file pone.0123345.s002.tiff]

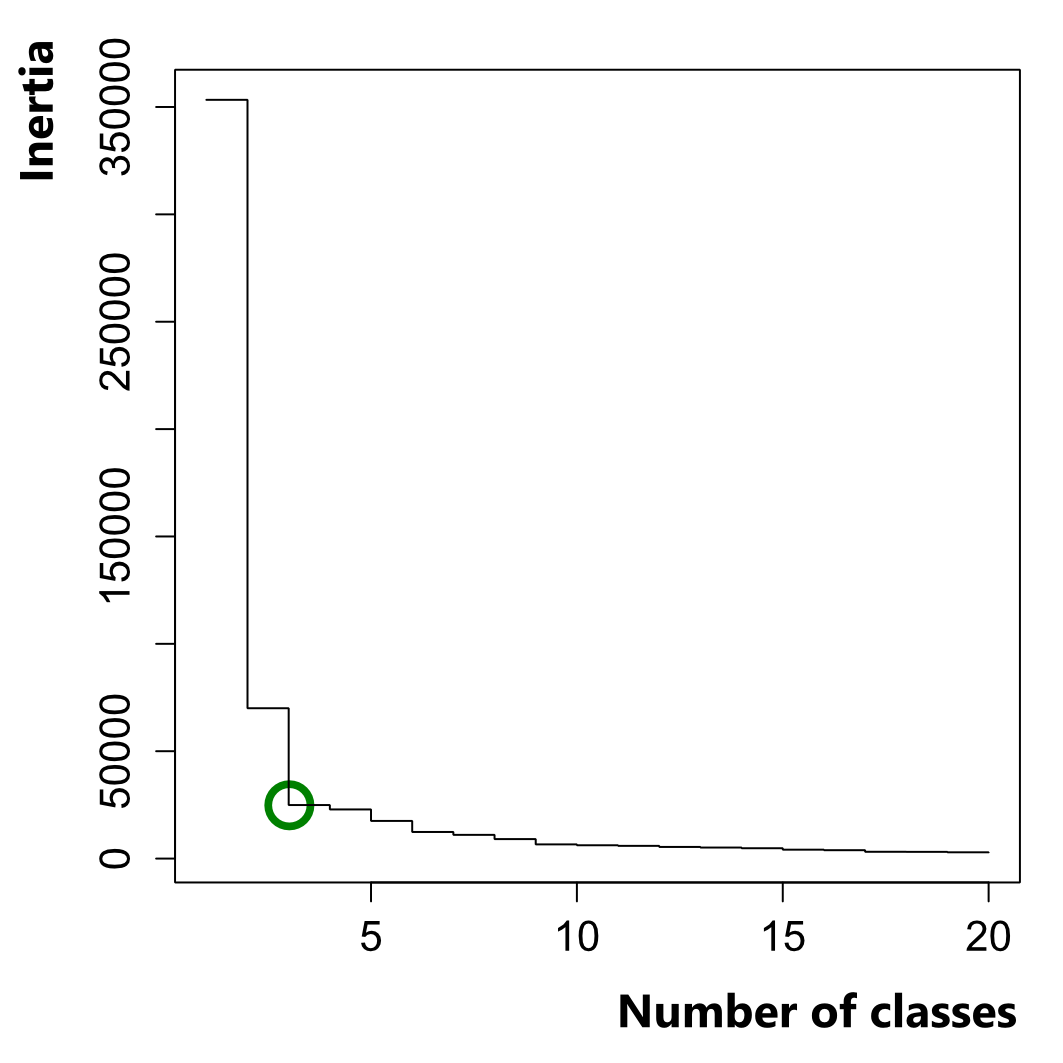

Supplement: S2 Fig — (TIFF) [file pone.0123345.s003.tiff]

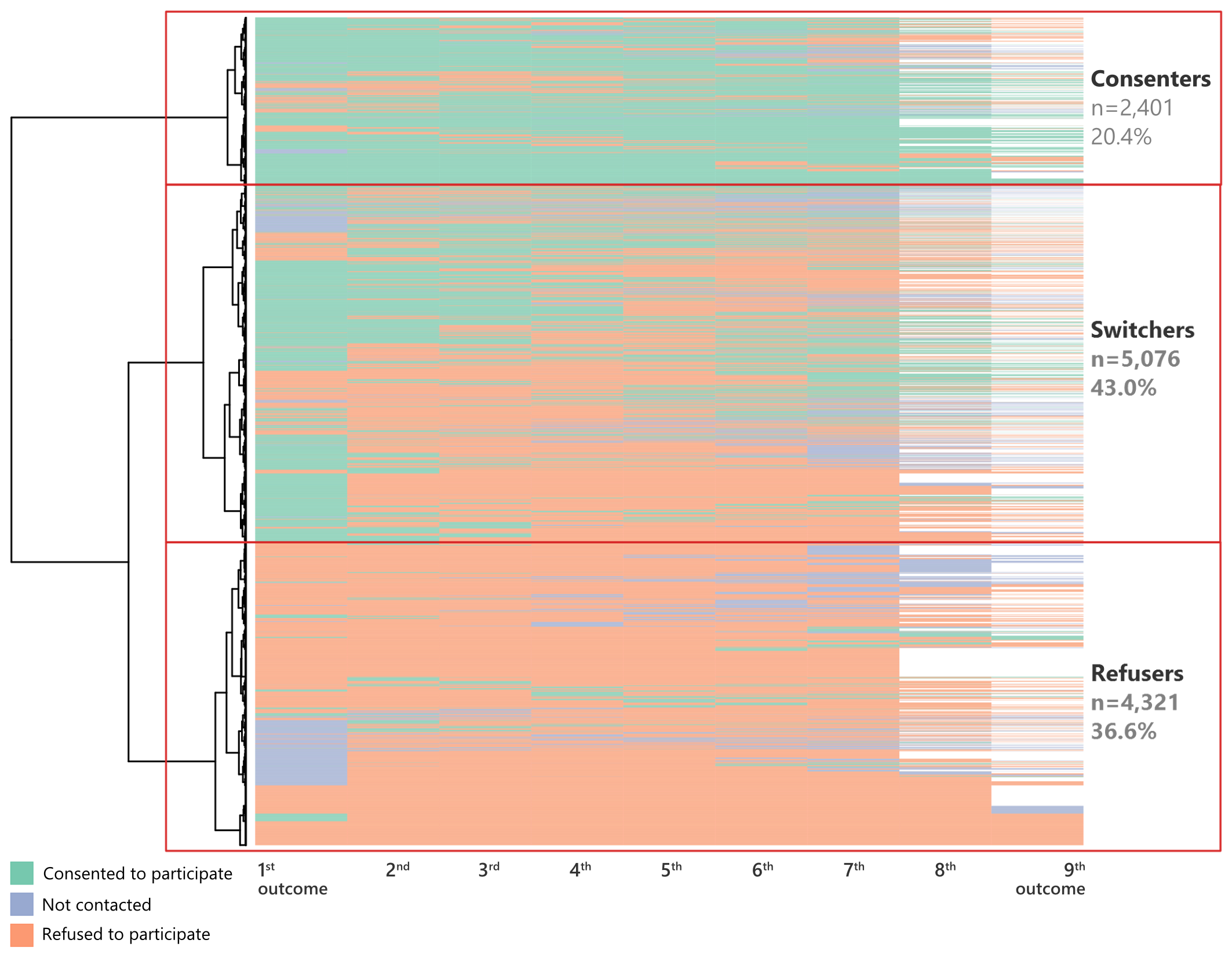

Supplement: S3 Fig — A dendrogram is a tree diagram illustrating the arrangement of the participation sequences produced by hierarchical clustering. Partition is obtained by cutting the dendrogram at a specific height and is represented by red rectangles. Profiles have been named according to their participation pattern (see Fig 5). (TIFF) [file pone.0123345.s004.tiff]
